# Younger and Late Middle-Aged Adults Exhibit Different Patterns of Cognitive-Motor Interference During Locomotor Adaptation, With No Disruption of Savings

**DOI:** 10.3389/fnagi.2021.729284

**Published:** 2021-11-26

**Authors:** Cristina Rossi, Ryan T. Roemmich, Nicolas Schweighofer, Amy J. Bastian, Kristan A. Leech

**Affiliations:** ^1^Center for Movement Studies, Kennedy Krieger Institute, Baltimore, MD, United States; ^2^Department of Biomedical Engineering, Johns Hopkins University School of Medicine, Baltimore, MD, United States; ^3^Department of Physical Medicine and Rehabilitation, Johns Hopkins University School of Medicine, Baltimore, MD, United States; ^4^Division of Biokinesiology and Physical Therapy, University of Southern California, Los Angeles, CA, United States; ^5^Department of Neuroscience, Johns Hopkins University School of Medicine, Baltimore, MD, United States

**Keywords:** adaptation, savings, cognition, dual-task, aging

## Abstract

It has been proposed that motor adaptation and subsequent savings (or faster relearning) of an adapted movement pattern are mediated by cognitive processes. Here, we evaluated the pattern of cognitive-motor interference that emerges when young and late middle-aged adults perform an executive working memory task during locomotor adaptation. We also asked if this interferes with savings of a newly learned walking pattern, as has been suggested by a study of reaching adaptation. We studied split-belt treadmill adaptation and savings in young (21 ± 2 y/o) and late middle-aged (56 ± 6 y/o) adults with or without a secondary 2-back task during adaptation. We found that young adults showed similar performance on the 2-back task during baseline and adaptation, suggesting no effect of the dual-task on cognitive performance; however, dual-tasking interfered with adaptation over the first few steps. Conversely, dual-tasking caused a decrement in cognitive performance in late middle-aged adults with no effect on adaptation. To determine if this effect was specific to adaptation, we also evaluated dual-task interference in late middle-aged adults that dual-tasked while walking in a complex environment that did not induce motor adaptation. This group exhibited less cognitive-motor interference than late middle-aged adults who dual-tasked during adaptation. Savings was unaffected by dual-tasking in both young and late middle-aged adults, which may indicate different underlying mechanisms for savings of reaching and walking. Collectively, our findings reveal an age-dependent effect of cognitive-motor interference during dual-task locomotor adaptation and no effect of dual-tasking on savings, regardless of age. Young adults maintain cognitive performance and show a mild decrement in locomotor adaptation, while late middle-aged adults adapt locomotion at the expense of cognitive performance.

## Introduction

Sensorimotor adaptation is a form of motor learning that occurs in response to a sensory prediction error caused by a sustained, predictable perturbation in the environment (Martin et al., 1996; Bastian, 2008; Shadmehr et al., 2010). That is, movements are adapted or updated over time to reduce the sensory prediction error and improve performance in the perturbed environment. Sensorimotor adaptation has traditionally been considered to be a primarily cerebellum-dependent process (Morton and Bastian, 2004; Tseng et al., 2007) that occurs automatically and implicitly. However, mounting evidence suggests that frontally mediated cognitive processes also contribute to sensorimotor adaptation-based learning (Taylor and Ivry, 2012, 2014).

Largely, evidence for a contribution of cognitive processes to sensorimotor adaptation comes from studies of upper extremity movements (for review in Taylor and Ivry, 2014; McDougle et al., 2016). This body of work demonstrates that, in addition to an implicit movement recalibration in response to a perturbation (e.g., a visuomotor rotation of reach angle), participants develop an explicit strategy to voluntarily adjust or aim their reaching movements to consciously counteract the perturbation. The development of these explicit movement strategies is thought to involve cognitive processes, such as executive control (Taylor and Thoroughman, 2008) and working memory (Seidler et al., 2010), which are mediated by structures in the prefrontal cortex (for review in Taylor and Ivry, 2012, 2014).

Cognitive processes may also contribute to sensorimotor adaptation during walking. However, given the relatively automatic nature of walking (it does not often require attention or an explicit strategy to “aim” our feet), the evidence for this is less clear. We and others have shown that young adults exhibit some degree of cognitive-motor interference when they simultaneously engage in a cognitive task while learning to walk on split-belt treadmill (Malone and Bastian, 2010; Sawers et al., 2013; Vervoort et al., 2019; Hinton et al., 2020). Split-belt treadmill walking is a well-studied locomotor adaptation paradigm traditionally thought to drive automatic, implicit adaptation of the walking pattern in response to the sensory prediction error induced by the right and left treadmill belts moving at different speeds (i.e., a mechanical perturbation). The presence of cognitive-motor interference during a dual-task locomotor adaptation paradigm suggests that neural resources between the cognitive task and locomotor adaptation are shared (Pashler and Johnston, 1998).

Interestingly, cognitive processes have also been proposed to contribute to the phenomenon of savings or faster re-learning of an upper extremity movement with re-exposure to the same perturbation (Morehead et al., 2015; Coltman et al., 2019). For example, Morehead et al. demonstrated that when participants are re-exposed to the same visuomotor learning task, they recognize the perturbation and re-engage an explicit aiming strategy to counteract the perturbation faster (Morehead et al., 2015). Our previous work has suggested that some degree of cognitive processing may also contribute to locomotor savings. Specifically, we have shown that the magnitude of savings is related to a participant’s ability to explicitly recall the split-belt perturbation size (Roemmich and Bastian, 2015). While this highlights a potential role for cognitive processes in locomotor savings, it is unclear how increasing the cognitive load during adaptation through a dual-task paradigm influences savings during walking.

Here we investigated: (1) the cognitive-motor interference pattern during a dual-task locomotor adaptation paradigm and (2) the impact of an increased cognitive load during adaptation on locomotor savings. We first tested these effects in two groups of neurotypical young adults; one dual-task group that completed an auditory 2-back task during locomotor adaptation and a single-task group that only adapted. Based on studies of savings in upper extremity movements (Morehead et al., 2015; Song and Bédard, 2015), we hypothesized that dual-tasking during adaptation would interfere with locomotor savings. However, given the differences in the neural control of upper and lower extremity movements, it was possible that we would not find interference with locomotor savings. Considering the well-documented effects of aging on motor adaptation (Bock, 2005; Seidler, 2006; Hardwick and Celnik, 2014; Sombric et al., 2017), fluid cognition (Harada et al., 2013; Spreng and Turner, 2019; Buckley and Pascual-Leone, 2020), and dual-tasking capacity (Smith et al., 2017), we then tested the same conditions in neurotypical late middle-aged adults. We expected to find a greater effect on savings in late middle-aged adults.

## Materials and Methods

### Participants

Twenty neurotypical young adults (aged 18–25 years, 13 female) and thirty neurotypical late middle-aged adults (aged 47–72 years, 21 female) volunteered to participate in the study. Specifically, two groups of 10 young participants (YASingle: 21.1 ± 2.6 years; YADual: 20.9 ± 1.4 years) took part in Experiment 1, and three groups of 10 late middle-aged participants (MASingle: 55.0 ± 5.4 years; MADual: 55.6 ± 7.0 years; MADualComplex: 57.7 ± 6.9 years) took part in Experiment 2. All participants were naïve to split-belt treadmill walking, prescreened for neurological disease or dysfunction, and provided informed written consent before participating. The protocol was approved by the Johns Hopkins Medicine Institutional Review Board.

### Treadmill Walking

**FIGURE 1:**
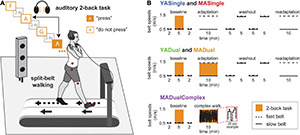
Experimental setup and paradigm. **(A)** Setup for walking and 2-back tasks. Participants walked on the split-belt treadmill with one belt (dashed arrow) moving faster than the other belt (solid arrow), and kinematics were recorded using markers positioned as shown (red circles). In the 2-back task, participants heard a string of letters through headphones, and were tasked to press a mouse button every time a letter was the same as 2 letters before. In the example shown, the participant should press the button on the second presentation of letter “A.” **(B)** Protocol for groups in Experiment 1 (YASingle and YADual) and Experiment 2 (MASingle, MADual, MADualComplex). Black lines show treadmill belt speeds throughout the experiment (solid: slow; dashed: fast). Orange blocks indicate phases when participants performed the 2-back task.

Participants walked on a custom-built split-belt treadmill (Woodway, Waukesha, WI, United States) that has two belts each driven by an independently controlled motor. We controlled the treadmill motors using a custom-written Python program. To start, participants stood in the middle of the treadmill with one foot on each belt. A 12-inch-tall partition was placed lengthwise between the belts to promote safety and prohibit stepping on both belts simultaneously but did not otherwise interfere with walking. Participants wore a safety harness suspended from the ceiling that did not provide body weight support. We informed the participants when the treadmill was about to start or stop but not of the speed of the belts. Immediately prior to starting the treadmill for all walking blocks, participants held on to a horizontal handrail with one hand and a computer mouse (to complete the cognitive task described below) with the other hand. Participants were instructed to release the rail and cross their arms as soon as the treadmill started moving (as depicted in Figure 1A). This was necessary to prevent blocking of the active marker set used for motion capture. Participants were also reminded to not look down at their feet or the treadmill during the experiment. The treadmill was stopped briefly (<1 min) between each walking block (e.g., baseline, adaptation, washout, etc.).

### Cognitive Task

At multiple time points in the experiment, participants completed a 2-back task. The 2-back task is a well-established cognitive task that taxes executive working memory (Kirchner, 1958) and engages multiple areas in the prefrontal cortex (Owen et al., 2005; Suzuki et al., 2018). It requires participants to attend to a string of stimuli (e.g., letters of the alphabet) and respond as quickly as possible when a stimulus is presented that is the same as the stimulus two positions prior. We chose to use the 2-back version of the n-back task based on the original n-back study from Kirchner (1958), which shows that 2-back provides the optimal level of cognitive challenge for both younger and older adults. Furthermore, pilot testing with different cognitive loadings (0-, 2-, or 3-back) suggested that the 2-back version would provide the largest dynamic range in our metric of interest (error rate), so that we would be able to capture any change – improvement or decline – in this metric due to cognitive-motor interference. Auditory letter stimuli were presented through headphones with an interstimulus interval of 2.4 s. Participants were instructed to listen to the string of letters and press the left-click button on a standard computer mouse (held in their dominant hand) each time they heard a letter that was the same as the letter that was stated two letters before it in the letter string (illustrated in Figure 1A). Hereafter, we will refer to these letters as “press” stimuli, and to letters different than the letter stated 2 letters before (such that participants should not press the mouse button) as “do not press” stimuli.

Unique random letter strings were generated for each testing block (i.e., standing, baseline, adaptation) for every participant using a custom written MATLAB code. To ensure that task difficulty was controlled between participants and testing blocks, we required that the random letter strings generated contain four possible press stimuli for each minute of the testing block. For example, within one minute of auditory 2-back testing, twenty-five letter stimuli were presented, four of which were a press stimulus. All participants were told “how quickly you respond and how accurate you are will be accounted for in your performance score – this means that an inaccurate response will count against you.” Regardless of group assignment, all participants had to perform the 2-back task in standing and baseline walking. When participants were required to dual-task (i.e., 2-back + walking), they were instructed to prioritize performance in the cognitive task; specifically, they were instructed to “try your best to do as well as you did on the cognitive task as you did while you were just standing on the treadmill.”

### Experimental Protocols

Experimental protocols for each group are shown in Figure 1B; note that the paradigm was identical between groups YASingle and MASingle, and between groups YADual and MADual. Baseline testing was consistent across all groups in Experiments 1 and 2. Participants initially performed the 2-back task during standing. Specifically, they were first given a 2-min practice 2-back task to ensure that they understood the instructions and could clearly hear the letter string. Participants then completed two standing 5-min 2-back tasks. This was followed by walking with the belts tied at 0.5 m/s for a total of 9 min. For the first 2 min of this block, participants walked on the treadmill without the 2-back task. For the next 5 min, they continued to walk while simultaneously performing the auditory 2-back task. Participants were given a 10-s warning before the start of the 2-back task. After completing the 2-back task, they walked for an additional 2 min.

For YASingle, YADual, MASingle and MADual groups, this was followed by walking with the belt speeds split in a 3:1 ratio (dominant leg 0.5 m/s and non-dominant leg 1.5 m/s) during a 10-min adaptation block (Figure 1B). Participants in the YADual and MADual groups adapted their walking patterns while simultaneously performing the auditory 2-back task, while participants in the YASingle and MASingle groups adapted their walking patterns without an additional cognitive task. After adaptation, all participants completed four 5-min tied-belt washout blocks that alternated between 0.5 and 1.5 m/s. All participants then completed a second adaptation block without a simultaneous cognitive task at the same belt speed configuration as the first adaptation block (readaptation; 10 min) to assess savings.

Because we found evidence of motor-related cognitive interference in the MADual group, we added another group to Experiment 2 to determine if the interference was specific to motor *adaptation*. The additional group (MADualComplex) performed the 2-back task while walking on the treadmill in an environment designed to be challenging and distracting but that would not induce motor adaptation. Specifically, after baseline, participants in the MADualComplex group walked for 10 min as the belt speeds remained tied but changed abruptly and randomly without warning (Figure 1B). This walking environment is not thought to induce sensorimotor adaptation because it does not introduce a predictable perturbation (Herzfeld et al., 2014). To create this environment, we generated a sequence of random belt speeds and times of exposure to each speed that were unique to each participant. The speeds were drawn from a uniform distribution between 0.5 and 1.5 m/s, and the times were drawn from a uniform distribution between 1 and 3 s. The minimum difference between each speed and the subsequent speed was constrained to be larger than 0.1 m/s to ensure that the environment would be complex enough. The average speed over the 10-min block was constrained to be 1 m/s to match the average speed of the MADual group who underwent motor adaptation.

### Data Collection

#### Motor Data

Kinematic data were collected at 100 Hz using Optotrak Certus motion capture hardware (Northern Digital, Waterloo, ON, Canada). Infrared-emitting active markers were placed bilaterally over the 5th metatarsal head, lateral malleolus, lateral femoral epicondyle, greater trochanter, iliac crest, and acromion process (Figure 1A). All participants remained on the treadmill throughout the duration of the testing session and wore comfortable walking shoes and form fitting clothing to reduce marker movement artifact.

#### Cognitive Data

During the 2-back testing blocks, each button press by the participants in response to a stimulus presentation was recorded and categorized as correct or incorrect. We also recorded the timing of each response relative to the onset of the stimulus presentation.

### Data and Statistical Analysis

Our collection of both motor and cognitive data allowed us to evaluate different patterns of potential cognitive-motor interference (Plummer et al., 2013). Specifically, measures of performance in the motor and cognitive domains allowed us to determine if simultaneous performance of an adaptive locomotor learning task and an executive working memory task led to: (1) no interference (performance does not change in either domain), (2) mutual interference (performance degrades in both domains), (3) cognitive-related motor interference (cognitive performance is stable while motor behavior is impacted), or (4) motor-related cognitive interference (motor behavior is unchanged while cognitive performance deteriorates). Details for the related data and statistical analyses are below.

#### Motor Data

For groups that adapted and readapted (YASingle, YADual, MASingle, and MADual), the primary motor outcome measure was step length asymmetry: (fast step length – slow step length)/(fast step length + slow step length). This metric adapts robustly during split-belt treadmill walking and is a commonly used behavioral marker for adaptive locomotor learning (e.g., Reisman et al., 2005, 2007; Hoogkamer et al., 2015; Roemmich and Bastian, 2015; Darter et al., 2018; Leech et al., 2018; Rossi et al., 2019; Mariscal et al., 2020). A value of zero step length asymmetry indicates stepping with symmetric step lengths. We calculated step length as the distance between the ankle markers along the anterior-posterior axis at heel-strike of each leg. Of note, we did not analyze a motor outcome measure for the group of late middle-aged adults that experienced random changes in tied-belt speeds (MADualComplex), as they were not exposed to split-belts and no motor adaptation was expected to occur.

To assess for cognitive-related motor interference, we compared step length asymmetry in adaptation, washout, and readaptation between the Single and Dual groups within each experiment. Similar to previously published work (e.g., Roemmich and Bastian, 2015; Vazquez et al., 2015; Leech et al., 2018), we characterized initial (strides 1–5), early (strides 6–30), and late (last 30 strides) epochs for both adaptation and readaptation by calculating the mean step length asymmetry within each epoch for each individual participant. We also characterized initial and early epochs of the washout phase using similar definitions. To compare each time epoch between the two groups, we performed a bootstrap analysis and computed confidence intervals (CI) of the difference between Single minus Dual group means (Efron and Tibshirani, 1994). Specifically, for each experiment and each time epoch, the analysis consisted of the following steps:

(1) we obtained 10,000 bootstrapped samples of 20 participants, with 10 participants resampled with replacement from each group independently;
(2) for each bootstrapped sample b, we computed the difference of the means between the Single and Dual groups, Δμ (*b*) = μ_*Single*_ (*b*) − μ_*Dual*_ (*b*), where μ_*Single*_ (*b*) is the mean step length asymmetry (for the time epoch of interest) of the 10 participants resampled from the Single group; μ_*Dual*_ (*b*) is analogous but from the Dual group (Efron and Tibshirani, 1994);
(3) we computed the 95% CI for the difference of means (i.e., with significance level α = 0.05);
(4) if the epoch was significantly different between the groups (as defined below), we adjusted the CI to account for multiple comparisons. We specifically corrected the significance level to $\alpha =\frac{R}{m}*0.05$, where R is the total number of epochs statistically different between the groups, and *m* = 10 is the total number of comparisons evaluated for the step length asymmetry difference measure (Benjamini and Yekutieli, 2005).

For each epoch, we interpreted step length asymmetry to be significantly different between the Single and Dual groups if the CI did not overlap zero (Efron and Tibshirani, 1994; DiCiccio and Efron, 1996; Cumming and Finch, 2005).

We finally investigated whether dual-tasking in adaptation affected savings. A metric of savings was computed for each participant as the difference in step length asymmetry between readaptation minus adaptation (similar to Mawase et al., 2014; Morehead et al., 2015; Song and Bédard, 2015) at the initial (strides 1–5) and early (strides 6–30) time epochs. We used a bootstrap analysis analogous to that described above to assess whether savings differed between Single and Dual groups in each experiment (with *m* = 2 for the correction for multiple comparisons).

To ensure a fair comparison of adaptation, readaptation, and savings between Single and Dual groups in each experiment, we also evaluated whether the groups were similar in baseline and end of washout. We compared step length asymmetry between Single and Dual groups in each experiment in baseline (last single-task block of 2 min), and late washout (last 30 strides before readaptation). We performed bootstrap analyses for these measures that were analogous to that described for adaptation, washout, and readaptation.

In addition to our primary metric of step length asymmetry, we also computed double support asymmetry and limb excursion asymmetry because previous studies of dual-tasking in locomotor adaptation reported evidence of cognitive-related motor interference in these metrics (Conradsson et al., 2019; Vervoort et al., 2019). The analysis for these metrics was analogous to that carried out for step length asymmetry, and details are reported in the Supplementary Methods.

In Experiment 1, we found that step length asymmetry was more negative (i.e., more asymmetric) in dual-tasking young adults (YADual) compared to single-tasking young adults (YASingle) in the initial epoch (average of strides 1–5) of both adaptation and readaptation. While we chose our primary analysis to be consistent with a large number of walking adaptation studies (e.g., Reisman et al., 2007; Hoogkamer et al., 2015; Roemmich and Bastian, 2015; Darter et al., 2018; Leech et al., 2018; Rossi et al., 2019; Mariscal et al., 2020), binning the data relies on assumptions about stride selection and it reduces resolution in the data by averaging across strides within an epoch. Furthermore, it does not dissect how cognitive-related motor interference affects the fast versus slow learning components which are thought to underly locomotor adaptation (Mawase et al., 2013; Roemmich et al., 2016; Rashid et al., 2020), and these components have been suggested to engage cognitive resources to different extents, at least in reaching adaptation (McDougle et al., 2015). To overcome these limitations, we performed a supplementary analysis where we modeled step length asymmetry during adaptation and readaptation using double-exponential functions (similar to Musselman et al., 2011; Vasudevan et al., 2011; Mawase et al., 2013; Rashid et al., 2020). This analysis is described in the Supplementary Methods.

#### Cognitive Data

For groups that performed the 2-back task in both baseline and adaptation/complex walking (YADual, MADual, and MADualComplex), we computed metrics for accuracy and speed to evaluate performance in the task. To assess accuracy with the task, we quantified error rate with press and do not press stimuli separately using participants’ binomial response data. Incorrect omissions to press stimuli and incorrect responses to do not press stimuli were considered errors and assigned a value of 1. Correct responses to press stimuli and correct omissions to do not press stimuli were instead given a value of 0. We fit the binomial data separately for the baseline 2-back task and the adaptation/complex walking 2-back task, using logistic regression models of the form: Error ∼ Stimulus. We defined Stimulus as the stimulus number within each phase (i.e., Stimulus ranged from 0 to 124 in baseline and from 0 to 249 in adaptation). We also calculated reaction time (RT) for correct press stimuli as the time of response relative to the onset of stimulus presentation. To fit the RT data, we used a linear regression model of the form: RT ∼ Stimulus, where Stimulus was defined as above.

We fit the models for the three measures of interest to each group separately. We used a bootstrapping procedure similar to that described for the motor analysis: we obtained 10,000 bootstrap samples of 10 participants, resampled with replacement from a single group, and fit the logistic or linear model to the error rate or RT of each sample. We then used the bootstrapped model fits of our measures to assess whether performance was worse in adaptation/complex walking as compared to baseline, which would be an indicator of motor-related cognitive interference. Specifically, for each of our three metrics (error rate in press stimuli, error rate in do not press stimuli, and RT), we computed the CI of the difference between the start of adaptation (first stimulus) minus the end of baseline (last stimulus). Adaptation performance was deemed statistically worse than baseline performance if the bounds of the CI were greater than zero. For each cognitive metric, we also evaluated whether the slope of the logistic or linear fit was statistically different from zero. To do so, we computed the CI of the coefficient for Stimulus in our model using our bootstrapping procedure. If the CI did not overlap with zero, this would suggest that the error rate or RT measures changed significantly throughout baseline or adaptation. In particular, a significantly negative slope in adaptation would suggest that error rate or RT improved throughout adaptation.

We first computed all CIs using α=0.05 (i.e., 95% CI). We then adjusted for multiple comparisons using the analysis previously described for the motor data; for each of the cognitive measures, we set the total number of comparisons *m* = 3. For completeness, we also computed the 95% CI of the intercept of our models.

## Results

### Experiment 1: Dual-Tasking During Locomotor Adaptation Leads to Cognitive-Related Motor Interference Very Early in Adaptation and Readaptation in Young Adults, but Does Not Affect Locomotor Savings

The goals of Experiment 1 were (1) to identify the pattern of cognitive-motor interference in young adults who performed a cognitive task while simultaneously learning a new walking pattern through adaptation and (2) to determine the impact of dual-tasking during locomotor adaptation on savings of the newly learned walking pattern. We investigated whether dual-tasking affected: (1) cognitive task performance, (2) locomotor adaptation, and/or (3) locomotor savings. All metrics were evaluated through bootstrapping and are reported as mean [95% CI] *{corrected CI}*. Note that the means and CIs reported for the motor metrics refer to the *difference* in metric between YASingle minus YADual group (see section “Materials and Methods”).

**FIGURE 2:**
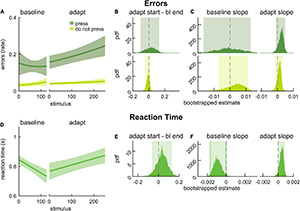
2-back performance during baseline walking and locomotor adaptation in young adults. **(A)** Logistic fits (mean and shaded SE) of the error rate in response to press stimuli (dark green) and do not press stimuli (light green) during an auditory 2-back task. **(B)** PDF of the differences in error rate estimates between the start of adaptation (first value) and the end of baseline (last value) from logistic model fits. **(C)** PDF of slope estimates for logistic fits of error rate in baseline and adaptation. **(D)** Linear fits (mean and shaded SE) of reaction time. **(E)** PDF of the differences in reaction time estimates between the start of adaptation (first value) and end of baseline (last value) estimated from linear model fits. **(F)** PDF of slope estimates for linear fits of reaction time in baseline and adaptation. Lighter shaded areas of PDF plots indicate the 95% CI; when applicable, darker shaded areas of PDF plots indicate the correction to the CI for 3 comparisons. SEs, PDFs, and CIs were obtained through bootstrapping. Cognitive performance was unchanged in adaptation.

We first evaluated the impact of the dual-task locomotor adaptation paradigm in the cognitive domain (Figure 2). That is, we assessed whether there was evidence of motor-related cognitive interference. To do this, we evaluated error rate and RT on the 2-back task during baseline walking and locomotor adaptation. Figure 2A shows model fits for error rate in press stimuli (dark green) and error rate in do not press stimuli (light green). We found that the differences in error rate between the end of baseline and the start of adaptation were not statistically different than zero for either stimulus type (Figure 2B; press: 0.031 [–0.101 0.130], do not press: –0.008 [–0.049 0.022]). We further evaluated the slope of the error rates and found that error rates (in response to either stimuli) did not change significantly over the course of baseline walking or adaptation (Figure 2C, press baseline: –0.001 [–0.016 0.013], adaptation: 0.003 [–0.001 0.005], do not press baseline: 0.004 [–0.007 0.011], adaptation: 0.001 [–0.001 0.003]). Model fits of RT in response to press stimuli during baseline and adaptation are presented in Figure 2D. The difference in RT between the start of adaptation and the end of baseline was not different from zero (Figure 2E, 0.046 [–0.064 0.128]). Participants’ RT did not change during baseline (Figure 2F; slope estimate for baseline: –0.0011 [–0.0016 –0.0001] *{–0.0018 0.0001}*) or as the new walking pattern was being learned during adaptation (slope estimate for adaptation: 0.0005 [–0.0001 0.0009]). Taken together, these results suggest that 2-back task performance was not affected by locomotor adaptation – young adults did not exhibit motor-related cognitive interference. Intercept values for the cognitive performance model fits can be found in Supplementary Table 1, and the results reported here (and corrected significance levels) are summarized in Supplementary Table 2.

**FIGURE 3:**
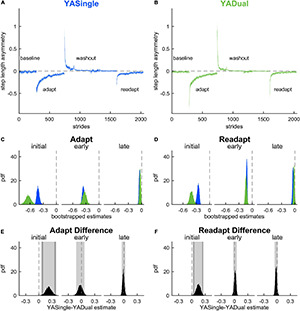
Motor adaptation and readaptation in single- and dual-tasking young adults. Step length asymmetry time course (mean and shaded SE) for **(A)** single-tasking (YASingle, blue) and **(B)** dual-tasking (YADual, green) young adults. **(C)** PDF of adaptation time epochs: initial (strides 1–5), early (strides 6–30), late (last 30 strides). **(D)** PDF of the same time epochs in readaptation. **(E)** PDF of the difference of the means between YASingle and YADual for adaptation time epochs. **(F)** PDF of the difference of the means between YASingle and YADual for readaptation time epochs. Lighter shaded areas of PDF plots indicate the 95% CI; when applicable, darker shaded areas of PDF plots indicate the correction to the CI for 10 comparisons. PDFs and CIs were obtained through bootstrapping. Initial adaptation and readaptation were more negative in dual- than single-tasking young adults.

We then assessed whether the motor behavior was affected by dual-tasking during locomotor adaptation (Figure 3) to determine if YADual exhibited cognitive-related motor interference. Figures 3A–D show the time course of step length asymmetry changes throughout the paradigm for the YASingle and YADual groups. To fairly assess adaptation and readaptation, we first ensured that both groups walked similarly at baseline (0.008 [–0.031 0.052]) and washed out to a similar extent (late washout: 0.005 [–0.010 0.024]).

We then compared adaptation behavior between the groups (Figure 3E) and found that dual-tasking young adults exhibited transient cognitive-related motor interference in the rapid step length asymmetry correction that occurs during the first 5 strides of adaptation (i.e., during the initial epoch). Specifically, YADual was initially more perturbed than YASingle (initial adaptation: 0.224 [0.098 0.352] *{0.061 0.392}*). This difference quickly dissipated as the stepping behaviors between the groups were not statistically different during early adaptation (–0.026 [–0.122 0.069]) or late adaptation (–0.011 [–0.051 0.031]) epochs. Both groups also exhibited similar aftereffects in step length asymmetry during washout (initial washout: –0.165 [–0.482 0.137], early washout: 0.026 [–0.085 0.128]). When comparing readaptation behavior between the groups (Figure 3F), we again found that YADual was initially more perturbed than YASingle (initial readaptation: 0.148 [0.057 0.233] *{0.026 0.257}*) but then walked similarly throughout the rest of readaptation (early readaptation: 0.013 [–0.029 0.052], late readaptation: –0.023 [–0.059 0.015]). These results (and adjusted confidence levels) are summarized in Supplementary Table 3. Our secondary analysis revealed that cognitive-related motor interference affected the initial magnitude of the fast-learning component of locomotor adaptation, such that the initial rapid correction in step length asymmetry was lower in YADual compared to YASingle in both adaptation and readaptation (Supplementary Figures 1, 2). Surprisingly, we also found that the rate of this component was faster in YADual than YASingle in adaptation. Dual-tasking did not interfere with the slower adaptation component, which contributes to the ongoing corrections through later stages of adaptation.

When we compared limb excursion asymmetry between YASingle and YADual groups, we found that the groups differed during initial adaptation (Supplementary Table 4). Limb excursion asymmetry did not differ between the groups at any other time epoch in adaptation, washout, or readaptation. Furthermore, we found that double support asymmetry was not different between YASingle and YADual groups during any phase of the paradigm (Supplementary Table 5).

**FIGURE 4:**
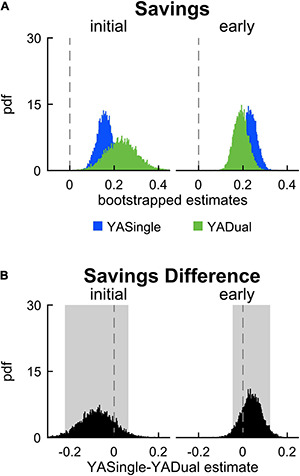
Savings in single- and dual-tasking young adults. **(A)** PDF of savings (difference in step length asymmetry between readapt and adapt) during the initial (strides 1–5) and early (strides 6–30) time epochs, for single-tasking (YASingle, blue) and dual-tasking (YADual, green) young adults. **(B)** PDF (and shaded 95% CI) of the difference of the means between YASingle and YADual for savings measures. PDFs and CIs were obtained through bootstrapping. Savings was not different between single- and dual-tasking young adults.

Lastly, we evaluated the impact of dual-tasking during adaptation (and the related cognitive-related motor interference) on locomotor savings of step length asymmetry. We measured savings by calculating the difference between step length asymmetry during adaptation and readaptation. Figure 4A displays the probability density functions for this metric during initial and early epochs for YADual and YASingle. We found that the performance of a cognitive task during adaptation did not interfere with locomotor savings – as both groups saved the new walking pattern similarly (Figure 4B; initial savings: –0.076 [–0.222 0.064], early savings: 0.039 [–0.046 0.123]). This is summarized in Supplementary Table 3. Our secondary analysis that evaluated savings in the fast and slow components of adaptation confirmed this result (Supplementary Figure 3). Finally, we found that savings of limb excursion asymmetry and double support asymmetry were not different between YASingle and YADual (Supplementary Tables 4, 5).

In conclusion, we found that dual-tasking during locomotor adaptation in young adults only leads to cognitive-related motor interference at very early stages of adaptation and readaptation. It did not lead to motor-related cognitive interference, and did not interfere with locomotor savings.

### Experiment 2: Dual-Tasking During Locomotor Adaptation Leads to Motor-Related Cognitive Interference in Late Middle-Aged Adults, but Does Not Affect Locomotor Savings

The primary goals of Experiment 2 were similar to those of Experiment 1, but here we evaluated late middle-aged adults to test the effects of age-related declines in cognitive processing (Tisserand et al., 2004; Reuter-Lorenz and Cappell, 2008; Cappell et al., 2010; Madden et al., 2010; Sala-Llonch et al., 2015) and the capacity for dual-tasking (Li et al., 2018; Vervoort et al., 2019). Motor and cognitive metrics were evaluated through bootstrapping and are reported as means [95% CI] *{corrected CI}*. As previously described, we report means and CIs of the *difference* in motor metrics between MASingle minus MADual group (see section “Materials and Methods”).

**FIGURE 5:**
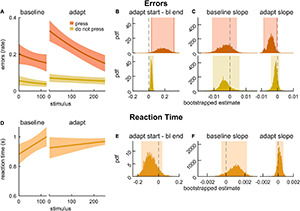
2-back performance during baseline walking and locomotor adaptation in late middle-aged adults. **(A)** Logistic fits (mean and shaded SE) of the error rate in response to press stimuli (dark orange) and do not press stimuli (yellow) during an auditory 2-back task. **(B)** PDF of the differences in error rate estimates between the start of adaptation (first value) and the end of baseline (last value) from logistic model fits. **(C)** PDF of slope estimates for logistic fits of error rate in baseline and adaptation. **(D)** Linear fits (mean and shaded SE) of reaction time. **(E)** PDF of the differences in reaction time estimates between the start of adaptation (first value) and end of baseline (last value) estimated from linear model fits. **(F)** PDF of slope estimates for linear fits of reaction time in baseline and adaptation. Lighter shaded areas of PDF plots indicate the 95% CI; when applicable, darker shaded areas of PDF plots indicate the correction to the CI for 3 comparisons. SEs, PDFs, and CIs were obtained through bootstrapping. Error rate with press and do not press stimuli declines with adaptation in late middle-aged adults, and error rate with press stimuli only improves throughout adaptation.

We first assessed whether the MADual group exhibited motor-related cognitive interference by evaluating changes in error rate and RT during the 2-back task between baseline walking and locomotor adaptation. Figure 5A depicts logistic model fits for error rate with press stimuli (dark orange) and error rate with do not press stimuli (yellow). In contrast to the results in Experiment 1, we found that late middle-aged adult participants exhibited motor-related cognitive interference. They made more errors at the start of adaptation relative to baseline walking in response to both types of stimuli (Figure 5B; press: 0.174 [0.040 0.309] *{0.028 0.320}*, do not press: 0.034 [0.016 0.054] *{0.011 0.058}*). We found that the error rate with both stimuli did not change throughout baseline (Figure 5C; baseline slope estimate for press: –0.002 [–0.011 0.004], do not press: –0.003 [–0.011 0.006]). Interestingly, errors with press stimuli during the adaptation block decreased as the new walking pattern was learned (Figure 5C; adapt slope estimate: –0.004 [–0.009 –0.001] *{–0.0088 –0.0002}*) whereas errors in response to do not press stimuli remained elevated over the course of motor adaptation (Figure 5C; adapt slope estimate: –0.001 [–0.004 0.001]). Model fits of RT in response to press stimuli during baseline and adaptation are presented in Figure 5D. The difference in RT between the start of adaptation and the end of baseline was not statistically different from zero (Figure 5E; –0.110 [–0.169 0.036]) nor were the slope estimates for baseline or adaptation (Figure 5F; baseline: 0.0012 [–0.0005 0.0022], adaptation: 0.0001 [–0.0003 0.0007]), indicating that accuracy was more affected by the dual-task. Intercept values for these cognitive performance fits are provided in Supplementary Table 1, and the results reported here (and adjusted confidence levels) are summarized in Supplementary Table 2. In sum, we found that the increase in error rate with press stimuli during adaptation was transient, and it decayed as participants adapted. On the contrary, the increase in error rate with do not press stimuli was longer-lasting and did not improve with adaptation.

**FIGURE 6:**
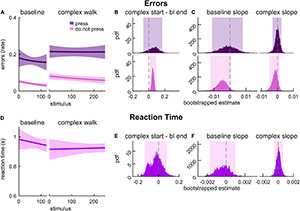
2-back performance during baseline and complex walking in late middle-aged adults. **(A)** Logistic fits (mean and shaded SE) of the error rate in response to press stimuli (dark purple) and do not press stimuli (pink) during an auditory 2-back task. **(B)** PDF of the differences in error rate estimates between the start of complex walking (first value) and the end of baseline (last value) from logistic model fits. **(C)** PDF of slope estimates for logistic fits of error rate in baseline and complex walking. **(D)** Linear fits (mean and shaded SE) of reaction time. **(E)** PDF of the differences in reaction time estimates between the start of complex walking (first value) and end of baseline (last value) estimated from linear model fits. **(F)** PDF of slope estimates for linear fits of reaction time in baseline and complex walking. Lighter shaded areas of PDF plots indicate the 95% CI; when applicable, darker shaded areas of PDF plots indicate the correction to the CI for 3 comparisons. SEs, PDFs, and CIs were obtained through bootstrapping. Error rate with do not press stimuli only declines with complex walking in late middle-aged adults.

We next aimed to understand the relationship between the motor-related cognitive interference observed in late middle-aged adults and cognitive processes that contribute to locomotor adaptation versus walking in a complex environment. To this end, we tested another group of late middle-aged adults that completed the 2-back task while walking on a treadmill as the speed changed randomly and abruptly (MADualComplex; Figure 1B). Figure 6A depicts the model fits for error rate in response to press stimuli (dark purple) and do not press stimuli (pink) for MADualComplex. We found that the error rate with press stimuli did not increase during complex walking relative to baseline (Figure 6B; 0.045 [–0.069 0.168]) and did not change over time (Figure 6C; slope estimate for baseline: –0.001 [–0.011 0.008], complex walking: 0.001 [–0.003 0.002]). However, the error rates with do not press stimuli were higher at the start of complex walking as compared to the end of baseline (Figure 6B; 0.051 [0.025 0.079] *{0.020 0.084}*) and remained elevated (Figure 6C; slope estimate for baseline: –0.005 [–0.012 0.001], complex walking: –0.001 [–0.005 0.001]). Model fits of RT in response to press stimuli during baseline and complex walking are presented in Figure 6D. The start of complex walking did not have an effect on RT relative to the end of baseline (Figure 6E, –0.043 [–0.135 0.081]). RT estimates were stable over time during both baseline and complex walking (Figure 6F; slope estimate for baseline: –0.0002 [–0.0017 0.0010], complex walking: 0.0001 [–0.0005 0.0006]). Intercept estimates for all model fits of these cognitive performance estimates are provided in Supplementary Table 1, and the results reported here (and adjusted confidence levels) are summarized in Supplementary Table 2. These results indicate that cognitive processes that contribute to the control of walking in complex environments (independent of adaptation) also mediate do not press stimuli 2-back performance whereas those that mediate accurate 2-back performance with press stimuli specifically overlap with cognitive processes involved in locomotor adaptation.

**FIGURE 7:**
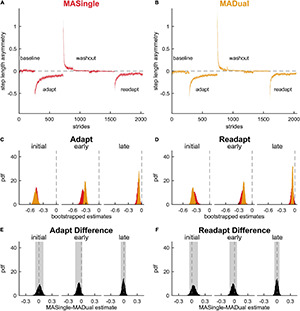
Motor adaptation and readaptation in single- and dual-tasking late middle-aged adults. Step length asymmetry time course (mean and shaded SE) for **(A)** single-tasking (MASingle, red) and **(B)** dual-tasking (MADual, orange) late middle-aged adults. **(C)** PDF of adaptation time epochs: initial (strides 1–5), early (strides 6–30), late (last 30 strides). **(D)** PDF of the same time epochs in readaptation. **(E)** PDF of the difference of the means between MASingle and MADual for adaptation time epochs. **(F)** PDF of the difference of the means between MASingle and MADual for readaptation time epochs. Lighter shaded areas of PDF plots indicate the 95% CI. PDFs and CIs are obtained through bootstrapping. Motor adaptation and readaptation are not significantly different between MASingle and MADual groups.

**FIGURE 8:**
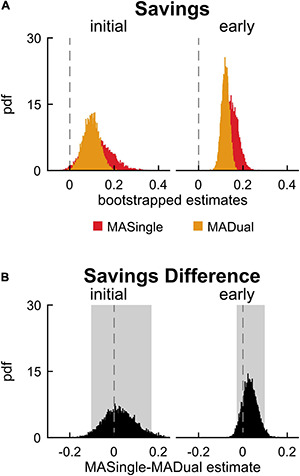
Savings in single- and dual-tasking late middle-aged adults. **(A)** PDF of savings (difference in step length asymmetry between readapt and adapt) during the initial (strides 1–5) and early (strides 6–30) time epochs, for single-tasking (MASingle, red) and dual-tasking (MADual, orange) late middle-aged adults. **(B)** PDF (and shaded 95% CI) of the difference of the means between MASingle and MADual for savings measures. PDFs and CIs are obtained through bootstrapping. Savings does not differ between MASingle and MADual groups.

Finally, we investigated how the motor behavior of late middle-aged adults was affected by the cognitive task in MADual participants relative to MASingle participants to determine if there was mutual interference in the cognitive and motor domains. Contrary to our hypothesis, we did not find cognitive-related motor interference in late middle-aged adults, nor interference with locomotor savings. Figures 7A–D display step length asymmetry throughout the experimental paradigm for both groups. There were no significant differences between the groups in step length asymmetry during baseline walking (0.0299 [0.0001 0.0597] *{–0.0138 0.0707}*) and washout (initial washout: –0.163 [–0.476 0.153]; early washout: 0.000 [–0.089 0.081]; late washout: –0.011 [–0.029 0.007]). Contrary to our hypothesis and previous work (Malone and Bastian, 2016), we found that dual-tasking during adaptation lead to no significant differences in step length asymmetry in adaptation (Figure 7E; initial adaptation: 0.012 [–0.083 0.103], early adaptation: –0.062 [–0.144 0.016], late adaptation: –0.011 [–0.071 0.046]), readaptation (Figure 7F; initial readaptation: 0.037 [–0.057 0.138], early readaptation: –0.030 [–0.114 0.057], late readaptation: –0.005 [–0.063 0.053]), or savings (Figure 8; initial savings: 0.025 [–0.103 0.168], early savings: 0.032 [–0.028 0.098]). These results and adjusted confidence levels are summarized in Supplementary Table 3. Also contrary to previous work (Conradsson et al., 2019; Vervoort et al., 2019), there were no differences between MASingle and MADual in double support asymmetry or limb excursion asymmetry during any phase of the paradigm, or in savings (Supplementary Tables 4, 5). In summary, contrary to our hypothesis we found that dual-tasking during locomotor adaptation in late middle-aged adults did not lead to cognitive-related motor interference nor interfered with savings, rather it only interfered with performance in the cognitive task.

## Discussion

In this study, we evaluated the pattern of cognitive-motor interference in young and late middle-aged neurotypical adults while they performed a cognitive task during split-belt treadmill adaptation. We also assessed whether a concurrent cognitive task during adaptation would affect locomotor savings. We found evidence of cognitive-motor interference in both groups, but the pattern of cognitive-motor interference during adaptation differed between young and late middle-aged adults in dual-task conditions. Young adults in the dual-task group exhibited *cognitive-related motor interference* very early in adaptation. That is, they walked more asymmetrically at the beginning of adaptation relative to those who adapted without a cognitive task, while their performance on the cognitive task during adaptation was unaffected. Despite the cognitive-motor interference observed, contrary to our hypothesis dual-tasking during adaptation did not interfere with the savings of a newly learned walking pattern in young adults. Interestingly, we found that late middle-aged adults exhibited a different pattern of cognitive-motor interference; specifically, *motor-related cognitive interference* during adaptation. There were no differences in how the single and dual-task late middle-aged adult groups learned or saved the new walking pattern, but the early stages of locomotor adaptation interfered with the participants’ accuracy on the cognitive task. To evaluate the dependence of this result on the engagement of cognitive processes during locomotor adaptation versus the control of walking in a novel, complex environment, we tested a third group of late middle-aged adults. This group performed the 2-back task while walking in an environment designed to be challenging but not induce adaptation. We found that this group exhibited less motor-related cognitive interference than the group that adapted. This suggests that the cognitive interference observed in late middle-aged adults may be due only in part to overlapping neural resources between the cognitive task and locomotor adaptation.

### Dual-Tasking During Adaptation Did Not Interfere With Locomotor Savings in Either Age Group

Savings of an adapted movement is defined as faster relearning upon re-exposure to the same perturbation (Martin et al., 1996; Kojima et al., 2004; Smith et al., 2006). Prior work suggests that cognitive processes mediate savings of adapted upper extremity movements (Morehead et al., 2015; Avraham et al., 2021). While indirect evidence suggests cognitive processes also contribute to savings of an adapted walking pattern (Roemmich and Bastian, 2015), we did not find that increasing the executive working memory load through a dual-task paradigm during adaptation interfered with locomotor savings in young or late middle-aged adults.

Savings is thought to reflect a person’s ability to recall a motor memory that was previously formed through motor adaptation (Lee and Schweighofer, 2009; Huang et al., 2011; Morehead et al., 2015; Orban De Xivry and Lefèvre, 2015; Roemmich and Bastian, 2015) and is captured as the difference in motor behavior between adaptation and readaptation (Mawase et al., 2014; Morehead et al., 2015; Song and Bédard, 2015). Though we found that locomotor savings was unaffected in both age groups, dual-tasking young adults walked more asymmetrically at the start of adaptation and readaptation than single-tasking young adults. These results indicate that dual-tasking during adaptation may interfere with how young adults adapt and form a locomotor memory without impacting their ability to save and recall that memory. This is consistent with previous studies in patient populations that suggest the neural processes that underlie motor adaptation and savings may be distinct (Marinelli et al., 2009; Bédard and Sanes, 2011; Leow et al., 2012). Interestingly, this concept is also reflected in psychological studies that demonstrate the formation and recall of episodic memories are mediated by distinct neural mechanisms (Fletcher et al., 1995; Roy et al., 2017). Work in this domain has also shown that dual-tasking impairs the formation, but not recall, of episodic memories (Craik et al., 1996; Naveh-Benjamin et al., 2000).

One other study has examined the effect of dual-tasking on savings of an adapted movement. In Song and Bédard (2015), different groups of young adults engaged in a reaching adaptation and readaptation task. One group only adapted, another dual-tasked during adaptation, and a third group dual-tasked during both adaptation and readaptation. They found that the group that dual-tasked during both adaptation and readaptation exhibited comparable savings to the group that adapted alone. In contrast to our results, they found that participants who dual-tasked only during adaptation exhibited less savings than the groups that adapted alone or dual-tasked in both adaptation and readaptation. The authors suggest these results indicate savings was not affected by the increased cognitive load of dual-tasking, but rather the environmental context cues provided by the dual-task paradigm during learning. That is, the group that dual-tasked during adaptation (but not readaptation) exhibited less savings due to adapting and readapting with different environmental context cues.

This conclusion is supported by previous studies of upper extremity adaptation and savings that indicate motor memories are best recalled when environmental context cues are the same during adaptation and readaptation (Lee and Schweighofer, 2009; Addou et al., 2011; Hirashima and Nozaki, 2012; Howard et al., 2013). Yet, here we found that locomotor savings was unaffected when participants engaged in a dual-task only during adaptation. A previous study demonstrated that locomotor savings is related to a participant’s ability to explicitly recall the magnitude of the treadmill perturbation using solely proprioceptive feedback from the legs (Roemmich and Bastian, 2015). Together with the result presented here, this suggests that proprioceptive context cues, rather than environmental context cues provided by a secondary cognitive task, may play a larger role in the modulation of locomotor savings.

### Dual-Tasking During Adaptation Led to Cognitive-Related Motor Interference Very Early in Adaptation in Young Adults

Though the increased cognitive load from the dual-task paradigm had no effect on locomotor savings, young adults did exhibit cognitive-related motor interference affecting step length asymmetry very early during adaptation. The cognitive-related motor interference affected the initial magnitude of the fast-learning component of adaptation, which is thought to contribute to the initial rapid corrections in movement errors. This outcome is similar to previous dual-task adaptation studies that demonstrate varying patterns of cognitive-motor interference at the beginning of adaptation that dissipates at later stages of adaptation (Malone and Bastian, 2010; Song and Bédard, 2015; Vervoort et al., 2019; Hinton et al., 2020). Dual-task interference early during adaptation is consistent with the working theory that cognitive processes contribute primarily to the initial correction in movement errors (Taylor et al., 2014; Neville and Cressman, 2018) and are attributed to the fast-learning component of motor adaptation (McDougle et al., 2015). This interference effect may be explained by competition for shared neural resources between the motor adaptation and cognitive tasks. Neuroimaging studies have shown that locomotor adaptation and the n-back tasks both engage several common brain areas, some of which are traditionally associated with one domain or the other. These include the cerebellum (adapt: Hinton et al., 2019, n-back: Satterthwaite et al., 2013), parietal cortex (adapt: Hinton et al., 2019, n-back: Satterthwaite et al., 2013; Braun et al., 2015) and cingulate cortex (adapt: Hinton et al., 2019, n-back: Drobyshevsky et al., 2006; for a review of n-back neural substrates, see Munro et al., 2018).

Of note, the time course and pattern of cognitive-motor interference during dual-task locomotor adaptation paradigms in young adults varies between studies. Variation in the cognitive tasks across studies may help to explain why some have found cognitive-motor interference through later stages of adaptation (Malone and Bastian, 2010; Sawers et al., 2013) than we found with an auditory 2-back task. Other studies have employed a visual vigilance task (Sawers et al., 2013) and an audiovisual mental tracking task (Malone and Bastian, 2010). Importantly, each of these involves different aspects of cognition or domains of executive function (e.g., sustained attention, information processing, delayed recall, executive working memory; Al-Yahya et al., 2011; Loaiza et al., 2011; Wilhelm et al., 2013; Bayot et al., 2018) and likely engages distinct neural networks (Kansaku et al., 2004; Collette et al., 2005, 2006; Langner and Eickhoff, 2013; Yaple et al., 2019). The longer-lasting cognitive-motor interference found with vigilance or mental tracking dual-tasks indicates the related neural networks may overlap with the processes that underlie locomotor adaptation more than those engaged with an executive working memory task. In addition to variations in the time course, we also found a different pattern of cognitive-motor interference than some previous studies of dual-tasking during locomotor adaptation in young adults. Specifically, we found that that the dual-task led to interference only in the motor domain while other studies report interference in only the cognitive domain (Sawers et al., 2013; Hinton et al., 2020). This may be because participants in these studies were not given prioritization instructions (Hinton et al., 2020) or were told to prioritize the motor task (Sawers et al., 2013), as instructions (or a lack thereof) are known to affect task prioritization (Yogev-Seligmann et al., 2010; Jansen et al., 2016).

### Dual-Tasking During Locomotor Adaptation Led to Motor-Related Cognitive Interference in Late Middle-Aged Adults

In contrast to young adults, late middle-aged adults exhibited motor-related cognitive interference when dual-tasking during locomotor adaptation. Contrary to our hypothesis, late middle-aged adults did not exhibit cognitive-related motor interference, suggesting that late middle-aged adults prioritized the motor task – despite the instructions to prioritize the cognitive task. There are two reasons why task prioritization may have differed in late middle-aged versus young adults. First, previous work shows that the ability to deliberately allocate cognitive resources to a specific task declines with age (Yogev-Seligmann et al., 2010). Secondly, studies of dual-tasking during walking demonstrate that task prioritization is impacted by not only by instructions (Yogev-Seligmann et al., 2010; Jansen et al., 2016), but also the estimated hazard posed by the walking conditions (reviewed in Yogev-Seligmann et al., 2012). Locomotor adaptation challenges a learner’s stability (Buurke et al., 2018; Darter et al., 2018) and may be perceived as a hazardous walking condition by older adults, who are at a higher risk of falling than younger adults (Tinetti and Williams, 1998; Zijlstra et al., 2007; Ambrose et al., 2013).

Despite these compelling reasons for motor task prioritization, previous studies have found dual-task locomotor adaptation paradigms lead to motor interference in late middle-aged (Malone and Bastian, 2016) or older adults (Conradsson et al., 2019; Vervoort et al., 2019). This may be because the motor tasks employed were perceived as less hazardous than the task we used here (the belt speed differences were smaller and the average belt speeds were slower), allowing the older participants to prioritize cognitive over motor performance. However, in these studies cognitive performance was either not measured (Malone and Bastian, 2016; Conradsson et al., 2019), or was evaluated in a group that included both younger and older participants (Vervoort et al., 2019), so a direct comparison to our results is difficult. While the pattern of cognitive-motor interference differs across studies, older adults are consistently found to exhibit more robust (Vervoort et al., 2019) or longer-lasting (compare Malone and Bastian, 2016; Conradsson et al., 2019 to Malone and Bastian, 2010; Hinton et al., 2020) dual-task interference than younger adults. Our data similarly suggest that dual-task interference in late middle-aged adults extends further into adaptation than that exhibited by young adults. This may be explained by aging related declines in the structure and function of relevant brain areas (e.g., prefrontal cortex, Tisserand et al., 2004; Raz et al., 2005; Madden et al., 2010; Sala-Llonch et al., 2015; parietal cortex, Tisserand et al., 2004; Madden et al., 2010; and cingulate, Tisserand et al., 2004; Sala-Llonch et al., 2015), which are associated with worsened performance in cognitive tasks (Tisserand et al., 2004; Madden et al., 2010; Sala-Llonch et al., 2015) and result in over recruitment of prefrontal cortex (Reuter-Lorenz and Cappell, 2008; Cappell et al., 2010). However, it should be noted that some of the aforementioned work was performed in older adults (Tisserand et al., 2004; Cappell et al., 2010; Madden et al., 2010; Conradsson et al., 2019; Vervoort et al., 2019), and future work is needed to further dissect neural underpinnings of the motor-related cognitive interference in late middle-aged adults (see section “Limitations”).

The results of Experiment 2 also suggest that cognitive-motor interference during split-belt walking in middle-aged and older adults, observed by us and others (Malone and Bastian, 2016; Conradsson et al., 2019; Vervoort et al., 2019), is related to interference with *both* sensorimotor adaptation processes and those that control walking in a complex environment. From previous work, it was unclear if we could entirely attribute a decrement in cognitive performance during locomotor adaptation to an interference with sensorimotor adaptation processes. This is because split-belt walking not only induces sensorimotor adaptation, but also challenges participants’ stability (Buurke et al., 2018; Darter et al., 2018), which can also interfere with cognitive task performance (Lindenberger et al., 2000; Clark, 2015; Li et al., 2018). Here we found that participants in the MADual group exhibited an increased error rate with both press and do not press 2-back stimuli at the start of adaptation (Figure 5B). However, the increased error rate with press stimuli dissipated as the new walking pattern was learned (Figure 5C; adapt slope). This suggests that this portion of the motor-related cognitive interference was related specifically to overlapping neural resources with locomotor adaptation. Interestingly, the error rate with the do not press stimuli remained elevated throughout the adaptation block in the MADual group. We also found this effect in the MADualComplex group (Figures 6B,C). Taken together, these results suggest that the lasting interference with do not press stimuli responses may be due an overlap with the processes that more generally mediate the control of walking in a complex environment. A potential explanation for this portion of the cognitive interference is due to the heightened level of arousal that can be elicited by walking in environments that induce postural instability (Sibley et al., 2010) and locomotor learning (Green et al., 2010; Sibley et al., 2010). In fact, heightened arousal has been associated with an increased likelihood of responding to n-back task stimuli without regard for accuracy (Bottenheft et al., 2021).

While our data do support the idea that there is a shared neural resource between adaptation and working memory, it is yet unclear what specific domains of working memory may also be involved in locomotor adaptation. Working memory involves different domains of executive function (e.g., performance monitoring, rule encoding, processing speed, etc., Gajewski et al., 2018), each of which may correlate with different motor learning processes (Holland et al., 2019). Previous work has shown that *spatial* working memory is related to the rate of adaptation of upper extremity movements (Anguera et al., 2012; Christou et al., 2016; Vandevoorde and de Xivry, 2020) and is also linked to the use of explicit strategies during these tasks (Vandevoorde and de Xivry, 2020). While explicit strategies may play a role in locomotor adaptation driven through visual feedback (French et al., 2018), it is unlikely that they contribute to locomotor adaptation driven by a split-belt treadmill used here (Roemmich et al., 2016). Furthermore, a recent study by Sombric and Torres-Oviedo found that split-belt adaptation processes may be more related to cognitive switching ability (a domain of executive function) than to spatial working memory (Sombric and Torres-Oviedo, 2021). Yet, more work is needed to clarify the role of different domains of executive function in sensorimotor adaptation during walking. This may help identify sources of interindividual variability with sensorimotor adaptation-based learning paradigms.

### Limitations

While the current study provides new information on the effect of aging on cognitive-motor interference during locomotor adaptation, there are a few limitations that should be considered. First, the average age of the late middle-aged adult participants tested here was ∼55 years. The participants may not have been old enough to exhibit a robust mutual interference in both cognitive and motor domains as hypothesized, as aging related decline in the neural substrates involved in performing the 2-back task has been found to progress into later years of life (Yaple et al., 2019). Second, the sample sizes were relatively small (10 participants per group) and the results should be considered preliminary. It is possible that participants who enrolled in the study may not be fully representative of the young and late middle-aged populations of interest, or that we may not have had enough power to detect small differences between single- and dual-tasking groups. Future studies are needed that employ a wider age range and larger samples sizes.

## Conclusion

Here, we found that performing an executive working memory task during locomotor adaptation led to different patterns and time courses of cognitive-motor interference in young and late middle-aged adults. However, this did not interfere with locomotor savings in either age group. This supports existing evidence that cognitive processes contribute to sensorimotor adaptation during walking and dual-tasking during adaptation leads to a competition for shared neural resources. However, there remains lack of clarity about *what* cognitive processes or domains of executive function contribute to learning a new walking pattern through sensorimotor adaptation. Furthermore, because we found no effect of dual-task interference on savings, our data suggest that the cognitive processes that contribute to savings of an adapted walking pattern are likely distinct from those that contribute to learning a novel pattern initially.

## Data Availability Statement

The datasets presented in this study can be found in online repositories. The names of the repository/repositories and accession number(s) can be found below: Dryad Digital Repository: https://doi.org/10.5061/dryad.18931zcxm.

## Ethics Statement

The studies involving human participants were reviewed and approved by Johns Hopkins Institutional Review Board. The patients/participants provided their written informed consent to participate in this study.

## Author Contributions

KL and AB: study design and implementation. KL and CR: data acquisition and analysis. KL, CR, NS, RR, and AB: interpretation of the results, writing, and revision. All authors contributed to the article and approved the submitted version.

## Conflict of Interest

The authors declare that the research was conducted in the absence of any commercial or financial relationships that could be construed as a potential conflict of interest.

## Publisher’s Note

All claims expressed in this article are solely those of the authors and do not necessarily represent those of their affiliated organizations, or those of the publisher, the editors and the reviewers. Any product that may be evaluated in this article, or claim that may be made by its manufacturer, is not guaranteed or endorsed by the publisher.

## Abbreviations

RT: reaction time
CI: confidence interval
SE: standard error
PDF: probability density function.
