# Supplementary material for: Younger and Late Middle-Aged Adults Exhibit Different Patterns of Cognitive-Motor Interference During Locomotor Adaptation, With No Disruption of Savings
Source: Front Aging Neurosci. 2021 Nov 26;13:729284. doi: 10.3389/fnagi.2021.729284 (PMC8664558; doi:10.3389/fnagi.2021.729284)
Supplement: Supplementary file 1 [file Data_Sheet_1.pdf]

Supplementary Material

1 Supplementary Methods

1.1 Double support asymmetry and limb excursion asymmetry

For both Experiment 1 and 2, in addition to our primary measure of step length asymmetry, we computed metrics for double support asymmetry $\left(\frac{fast\ double\ support\ -slow\ double\ support}{fast\ double\ support\ +slow\ double\ support}\right)$ (Reisman et al., 2005; Conradsson et al., 2019; Vervoort et al., 2019) and limb excursion asymmetry $\left(\frac{fast\ limb\ excursion - slow\ limb\ excursion}{fast\ limb\ excursion + slow\ limb\ excursion}\right)$ (Vervoort et al., 2019). Double support was defined as the time during a step when both legs were in stance; specifically, we computed fast double support as the time between heel strike of the slow leg and subsequent toe off of the fast leg, and vice versa for slow double support. Limb excursion was defined as the anterior-posterior distance travelled by the ankle between heel strike and toe off of the same leg. We compared double support asymmetry and limb excursion asymmetry between single- and dual-tasking groups within the same experiment during baseline, adaptation, washout, and readaptation, using the same time epochs and bootstrapping analysis described in the main text regarding step length asymmetry.

1.2 Double-exponential fits of step length asymmetry

In Experiment 1, we also performed a secondary analysis of the step length asymmetry motor data and fitted double-exponential functions $\left(step\ length\ asymmetry = A_F \exp\left(-\frac{t}{\tau_F}\right) + A_S \exp\left(-\frac{t}{\tau_S}\right)\ \right)$ to adaptation and readaptation step length asymmetry curves (similar to Musselman et al., 2011; Vasudevan et al., 2011; Mawase et al., 2013; Rashid et al., 2020). We used the *fmincon* MATLAB function with initial conditions $A_F = -0.2$, $\tau_F = 10, A_S = -0.2, \tau_S = 100$, and we constrained $-1 \leq A_F \leq 0$, $-1 \leq A_s \leq 0$, and $0 \leq \tau_F \leq \tau_S \leq 5000$ and tightened *fmincon*'s Constraint Tolerance and Optimality Tolerance to 10^-20. We specifically performed a bootstrap analysis equivalent to that described in the main text and obtained confidence intervals for each of the 4 parameters from 10000 resamples of 10 participants. This was done separately for adaptation and readaptation, and for YASingle and YADual groups. For each of adaptation and readaptation phases, we statistically compared parameters between the YASingle and YADual groups using the same bootstrap procedure as that described for the main step length asymmetry analysis. We corrected for multiple comparisons with m=8. We also tested for differences in savings between the groups by computing savings metrics for each of the 4 parameters (defined as parameter value in readaptation, minus parameter value in adaptation), using the same bootstrap procedure as that described for the main analysis of savings. We corrected for multiple comparisons of savings metrics with m=4.

2 Supplementary Figures and Tables

2.1 Supplementary Figures

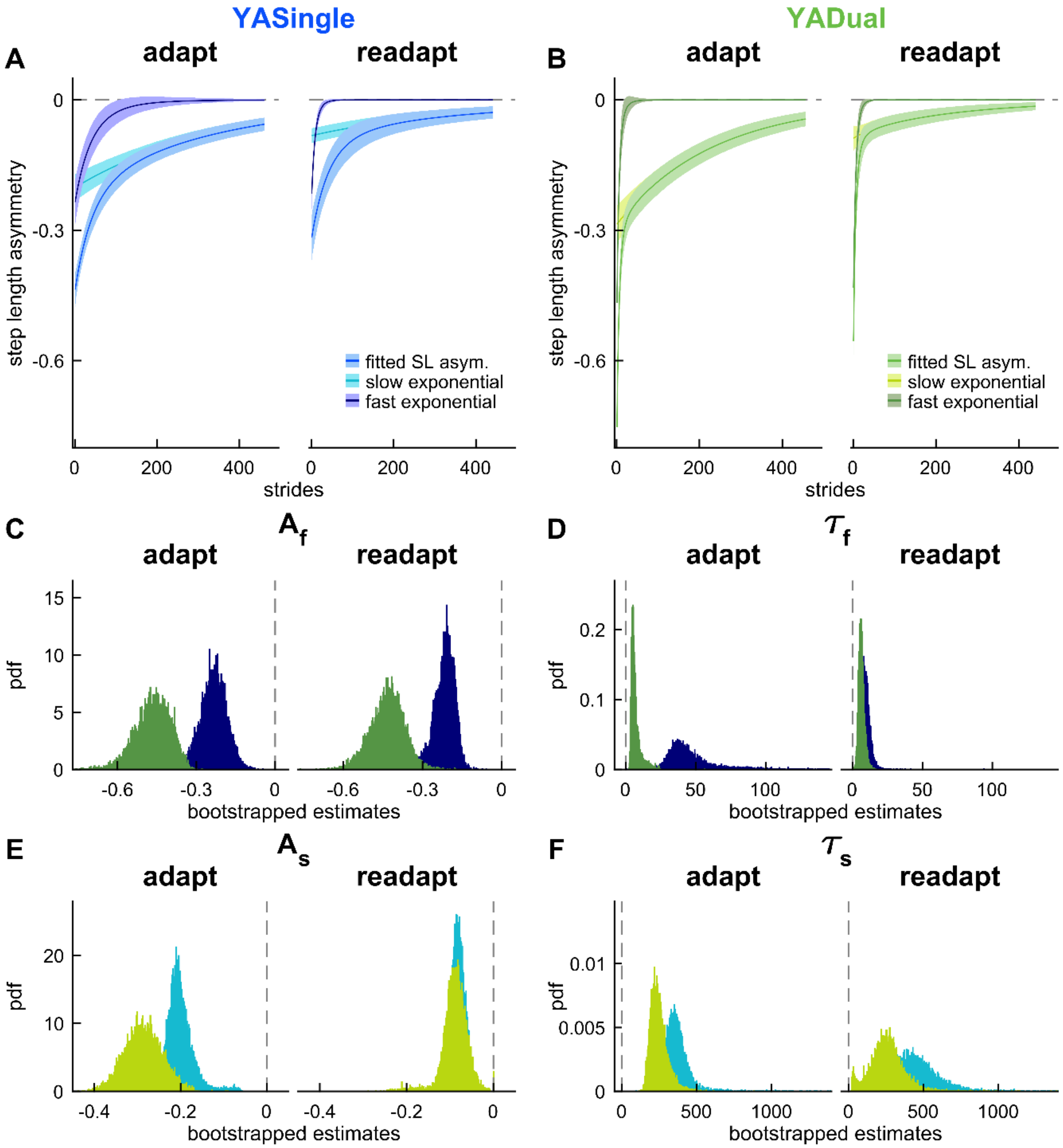

Supplementary Figure 1. Double exponential fits of step length asymmetry in adaptation and readaptation for YASingle and YADual. A-B) Time course of exponential fits. Shaded areas represent the bootstrapped SE. **C-F)** Probability density functions of the parameters A_f, τ_f, A_s, and τ_s estimated through bootstrapping.

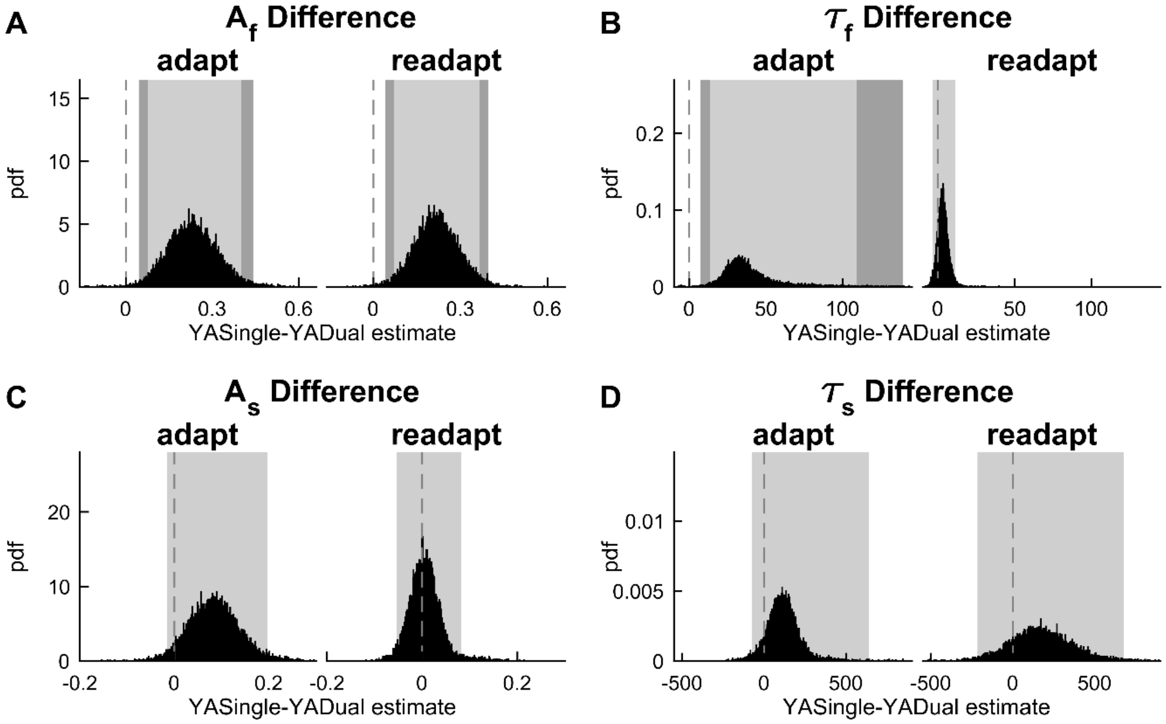

Supplementary Figure 2. Differences of parameter estimates between YASingle and YADual for the double exponential fits. The differences of parameter estimates (YASingle-YADual) for adaptation are as follows (mean [95% CI] *{corrected CI}*): A_f = 0.243 [0.075 0.401] *{0.044 0.441}*, τ_f = 37.095 [13.680 109.097] *{7.091 139.315}*, A_s = 0.080 [-0.016 0.197], τ_s = 114.465 [-75.179 636.026]. The differences of parameter estimates for readaptation are as follows (mean [95% CI] *{corrected CI}*): A_f = 0.217 [0.072 0.367] *{0.041 0.396}*, τ_f = 2.663 [-3.367 11.300], A_s = -0.002 [-0.053 0.082], τ_s = 148.491 [-215.806 677.629]. Lighter shaded areas of PDF plots indicate the 95% CI; when applicable, darker shaded areas of PDF plots indicate the correction to the CI for 8 comparisons (98.13% CI). PDFs and CIs are obtained through bootstrapping. No differences in savings of any parameter between the groups. A_f is more negative for YADual than for YASingle in both adaptation and readaptation.

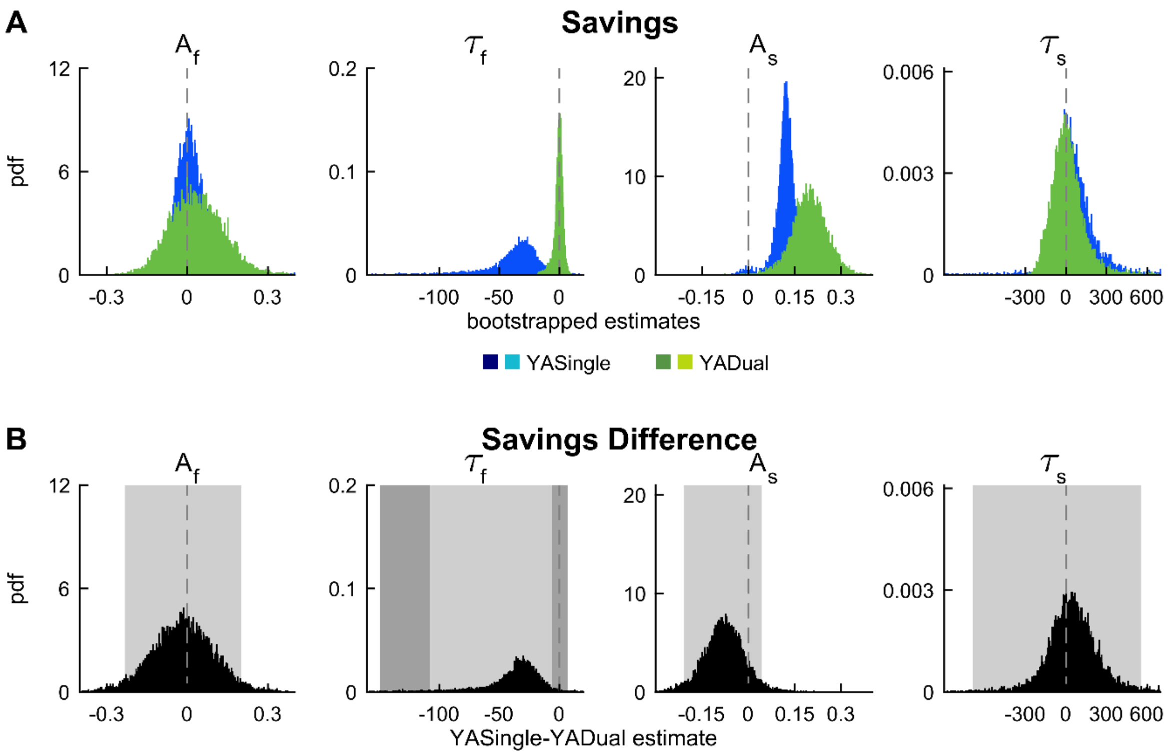

Supplementary Figure 3. Savings of double exponential fit parameters for YASingle and YADual. A) Probability density functions of parameters' savings, defined as parameter value in readaptation minus parameter value in adaptation. **B)** Differences of savings metrics between YASingle and YADual. Difference estimates are as follows (mean [95% CI] *{corrected CI}*): A_f = -0.026 [-0.233 0.200], τ_f = -34.432 [-107.733 -5.762] *{-149.233 6.908}*, A_s = -0.082 [-0.207 0.044], τ_s = 34.027 [-690.693 553.247]. Lighter shaded areas of PDF plots indicate the 95% CI; when applicable, darker shaded areas of PDF plots indicate the correction to the CI for 4 comparisons (98.75% CI). PDFs and CIs are obtained through bootstrapping. No differences in savings of any parameter between the groups.

2.2 Supplementary Tables

Supplementary Table 1. Intercept estimates for fits of error rate with press stimuli, error rate with do not press stimuli, and reaction time. Values are reported as mean [95% CI], where CIs are obtained through bootstrapping. The table is related to Figure 2, 5 and 6. *Note that Adapt refers to adaptation for YADual and MADual, but to complex walking for MADualComplex.

		**YADual**	**MADual**	**MADualComplex**
**Error rate** ***press***	**BL Intercept**	-1.835 [-3.088 -0.897]	-1.455 [-2.101 -0.756]	-1.597 [-2.228 -0.953]
	**Adapt* Intercept**	-1.694 [-2.337 -1.212]	-0.729 [-1.303 -0.287]	-1.384 [-1.803 -0.943]
**Error rate** ***do not press***	**BL Intercept**	-3.435 [-4.349 -2.771]	-2.840 [-4.638 -2.076]	-2.897 [-3.317 -2.568]
	**Adapt* Intercept**	-3.187 [-3.600 -2.842]	-2.574 [-3.423 -2.022]	-2.438 [-3.005 -2.025]
**Reaction time**	**BL Intercept**	0.854 [0.778 0.900]	0.888 [0.755 1.014]	0.977 [0.840 1.119]
	**Adapt* Intercept**	0.763 [0.660 0.879]	0.928 [0.783 1.064]	0.905 [0.779 1.048]

Supplementary Table 2. Slope estimates, and Adaptation (or Complex Walking) Start – Baseline End differences, for fits of error rate with press stimuli, error rate with do not press stimuli, and reaction time. Values are reported as mean [95% CI] *{corrected CI}*, where confidence levels were corrected to 98.33% [A] or 96.67% [B]. Significant values are highlighted in blue. The table is related to Figure 2, 5 and 6. *Note that Adapt refers to adaptation for YADual and MADual, but to complex walking for MADualComplex.

		YADual	MADual	MADualComplex
**Error rate *press***	**Adapt* – BL**	0.031 [-0.101 0.130]	**0.174 [0.040 0.309]** ***{0.028 0.320}*** [B]	0.045 [-0.069 0.168]
	**BL Slope**	-0.001 [-0.016 0.013]	-0.002 [-0.011 0.004]	-0.001 [-0.011 0.008]
	**Adapt* Slope**	0.003 [-0.001 0.005]	**-0.004 [-0.009 -0.001]** ***{-0.0088 -0.0002}*** [B]	0.001 [-0.003 0.002]
**Error rate *do not press***	**Adapt* – BL**	-0.008 [-0.049 0.022]	**0.034 [0.016 0.054]** ***{0.011 0.058}*** [A]	**0.051 [0.025 0.079]** ***{0.020 0.084}*** [A]
	**BL Slope**	0.004 [-0.007 0.011]	-0.003 [-0.011 0.006]	-0.005 [-0.012 0.001]
	**Adapt* Slope**	0.001 [-0.001 0.003]	-0.001 [-0.004 0.001]	-0.001 [-0.005 0.001]
**Reaction time**	**Adapt* – BL**	0.046 [-0.064 0.128]	-0.110 [-0.169 0.036]	-0.043 [-0.135 0.081]
	**BL Slope**	-0.0011 [-0.0016 -0.0001] *{-0.0018 0.0001}* [A]	0.0012 [-0.0005 0.0022]	-0.0002 [-0.0017 0.0010]
	**Adapt* Slope**	0.0005 [-0.0001 0.0009]	0.0001 [-0.0003 0.0007]	0.0001 [-0.0005 0.0006]

Supplementary Table 3. Difference in step length asymmetry and savings between Single and Dual groups in each experiment. Rows 1-10 report the mean step length asymmetry of the Single group, minus that of the Dual group, at selected time epochs. The last 2 rows represent the mean savings in step length asymmetry of the Single group, minus that of the Dual group. Values are reported as mean [95% CI] {*corrected CI*}; confidence levels were corrected to 99% [A] or 99.5% [B]. Significant values are highlighted in blue. The table is related to Figure 3, 4, 7, 8.

		YASingle-YADual	MASingle-MADual
**Baseline**	**Last 2 min**	0.008 [-0.031 0.052]	0.0299 [0.0001 0.0597] *{-0.0138 0.0707}* [B]
**Adaptation**	**Initial**	**0.224 [0.098 0.352]** ***{0.061 0.392}*** [A]	0.012 [-0.083 0.103]
	**Early**	-0.026 [-0.122 0.069]	-0.062 [-0.144 0.016]
	**Late**	-0.011 [-0.051 0.031]	-0.011 [-0.071 0.046]
**Washout**	**Initial**	-0.165 [-0.482 0.137]	-0.163 [-0.476 0.153]
	**Early**	0.026 [-0.085 0.128]	0.000 [-0.089 0.081]
	**Late**	0.005 [-0.010 0.024]	-0.011 [-0.029 0.007]
**Readaptation**	**Initial**	**0.148 [0.057 0.233]** ***{0.026 0.257}*** [A]	0.037 [-0.057 0.138]
	**Early**	0.013 [-0.029 0.052]	-0.030 [-0.114 0.057]
	**Late**	-0.023 [-0.059 0.015]	-0.005 [-0.063 0.053]
**Savings**	**Initial**	-0.076 [-0.222 0.064]	0.025 [-0.103 0.168]
	**Early**	0.039 [-0.046 0.123]	0.032 [-0.028 0.098]

Supplementary Table 4. Difference in limb excursion asymmetry and savings between Single and Dual groups in each experiment. Rows 1-10 report the mean limb excursion asymmetry of the Single group, minus that of the Dual group, at selected time epochs. The last 2 rows represent the mean savings of the Single group, minus that of the Dual group. Values are reported as mean [95% CI] *{corrected CI}*; confidence levels were corrected to 99% [A]. Significant values are highlighted in blue.

		YASingle-YADual	MASingle-MADual
**Baseline**	**Last 2 min**	0.005 [-0.003 0.014]	0.001 [-0.010 0.013]
**Adaptation**	**Initial**	**0.049 [0.012 0.089]** ***{0.002 0.103}*** [A]	0.031 [-0.003 0.066]
	**Early**	0.023 [-0.003 0.050]	0.002 [-0.023 0.027]
	**Late**	-0.015 [-0.032 0.003]	0.002 [-0.023 0.027]
**Washout**	**Initial**	-0.060 [-0.123 -0.006] *{-0.142 0.007}* [A]	-0.022 [-0.102 0.054]
	**Early**	-0.002 [-0.020 0.018]	0.004 [-0.015 0.022]
	**Late**	0.000 [-0.006 0.007]	0.000 [-0.007 0.007]
**Readaptation**	**Initial**	0.021 [-0.001 0.043]	0.015 [-0.019 0.051]
	**Early**	0.007 [-0.012 0.026]	0.011 [-0.025 0.049]
	**Late**	0.012 [-0.010 0.035]	0.002 [-0.022 0.025]
**Savings**	**Initial**	-0.028 [-0.070 0.008]	-0.016 [-0.048 0.017]
	**Early**	-0.016 [-0.039 0.005]	0.009 [-0.016 0.036]

Supplementary Table 5. Difference in double support asymmetry and savings between Single and Dual groups in each experiment. Rows 1-10 report the mean double support asymmetry of the Single group, minus that of the Dual group, at selected time epochs. The last 2 rows represent the mean savings of the Single group, minus that of the Dual group. Values are reported as mean [95% CI] *{corrected CI}*; confidence levels were corrected to 99.5% [A] or 99% [B].

		YASingle-YADual	MASingle-MADual
**Baseline**	**Last 2 min**	0.008<br>[-0.022 0.040]	0.018<br>[-0.024 0.058]
**Adaptation**	**Initial**	0.051<br>[-0.096 0.195]	-0.009<br>[-0.132 0.111]
	**Early**	0.074<br>[0.007 0.139]<br>*{-0.021 0.164}* [A]	0.100<br>[0.016 0.187]<br>*{-0.008 0.212}* [B]
	**Late**	-0.038<br>[-0.103 0.040]	0.064<br>[-0.042 0.178]
**Washout**	**Initial**	-0.072<br>[-0.219 0.068]	-0.045<br>[-0.180 0.085]
	**Early**	0.008<br>[-0.108 0.125]	-0.038<br>[-0.146 0.067]
	**Late**	0.001<br>[-0.051 0.046]	0.002<br>[-0.037 0.039]
**Readaptation**	**Initial**	0.045<br>[-0.038 0.132]	0.073<br>[-0.030 0.179]
	**Early**	-0.004<br>[-0.072 0.069]	0.095<br>[0.008 0.191]<br>*{-0.016 0.223}* [B]
	**Late**	-0.010<br>[-0.094 0.079]	0.074<br>[-0.033 0.190]
**Savings**	**Initial**	-0.006<br>[-0.133 0.107]	0.082<br>[-0.022 0.194]
	**Early**	-0.078<br>[-0.159 0.003]	-0.006<br>[-0.087 0.063]

3 Supplementary references

Conradsson, D., Hinton, D. C., and Paquette, C. (2019). The effects of dual-tasking on temporal gait adaptation and de-adaptation to the split-belt treadmill in older adults. *Exp. Gerontol.* 125, 110655. doi:10.1016/j.exger.2019.110655.

Mawase, F., Haizler, T., Bar-Haim, S., and Karniel, A. (2013). Kinetic adaptation during locomotion on a split-belt treadmill. *J. Neurophysiol.* 109, 2216–2227. doi:10.1152/jn.00938.2012.

Musselman, K. E., Patrick, S. K., Vasudevan, E. V. L., Bastian, A. J., and Yang, J. F. (2011). Unique characteristics of motor adaptation during walking in young children. *J. Neurophysiol.* 105, 2195–2203. doi:10.1152/jn.01002.2010.

Rashid, U., Kumari, N., Signal, N., Taylor, D., and Vandal, A. C. (2020). On nonlinear regression for trends in split-belt treadmill training. *Brain Sci.* 10, 737. doi:10.3390/brainsci10100737.

Reisman, D. S., Block, H. J., and Bastian, A. J. (2005). Interlimb coordination during locomotion: what can be adapted and stored? *J. Neurophysiol.* 94, 2403–2415. doi:10.1152/jn.00089.2005.

Vasudevan, E. V. L., Torres-Oviedo, G., Morton, S. M., Yang, J. F., and Bastian, A. J. (2011). Younger is not always better: development of locomotor adaptation from childhood to adulthood. *J. Neurosci.* 31, 3055–3065. doi:10.1523/JNEUROSCI.5781-10.2011.

Vervoort, D., Rob Den Otter, A., Buurke, T. J. W., Vuillerme, N., Hortobágyi, T., and Lamoth, C. J. C. (2019). Effects of aging and task prioritization on split-belt gait adaptation. *Front. Aging Neurosci.* 11, 10. doi:10.3389/fnagi.2019.00010.
